# Geniposide protected against cerebral ischemic injury through the anti-inflammatory effect via the NF-κB signaling pathway

**DOI:** 10.1515/tnsci-2022-0273

**Published:** 2023-06-09

**Authors:** Qian Sun, Xiangjian Zhang, Jingyi Fan, Lan Zhang, Hui Ji, Jing Xue, Cong Zhang, Rong Chen, Jing Zhao, Junmin Chen, Xiaoxia Liu, Degang Song

**Affiliations:** Department of Neurology, Second Hospital of Hebei Medical University, Shijiazhuang, Hebei, 050000, China; Hebei Collaborative Innovation Center for Cardio-Cerebrovascular Disease and Hebei Key Laboratory of Vascular Homeostasis, Shijiazhuang, Hebei, China; Department of Neurology, First Hospital of Qinhuangdao, Hebei, China

**Keywords:** MCAO, neuroinflammation, A20, TRAF6, NF-Kb

## Abstract

**Context:**

Accumulated evidence indicates that geniposide exhibits neuroprotective effects in ischemic stroke. However, the potential targets of geniposide remain unclear.

**Objective:**

We explore the potential targets of geniposide in ischemic stroke.

**Materials and methods:**

Adult male C57BL/6 mice were subjected to the middle cerebral artery occlusion (MCAO) model. Mice were randomly divided into five groups: Sham, MCAO, and geniposide-treated (i.p. twice daily for 3 days before MCAO) at doses of 25, 75, or 150 mg/kg. We first examined the neuroprotective effects of geniposide. Then, we further explored via biological information analysis and verified the underlying mechanism *in vivo* and *in vitro*.

**Results:**

In the current study, geniposide had no toxicity at concentrations of up to 150 mg/kg. Compared with the MCAO group, the 150 mg/kg group of geniposide significantly (*P* < 0.05) improved neurological deficits, brain edema (79.00 ± 0.57% vs 82.28 ± 0.53%), and infarct volume (45.10 ± 0.24% vs 54.73 ± 2.87%) at 24 h after MCAO. Biological information analysis showed that the protective effect was closely related to the inflammatory response. Geniposide suppressed interleukin-6 (IL-6) and inducible nitric oxide synthase (iNOS) expression in the brain homogenate, as measured by enzyme-linked immunosorbent assay (ELISA). Geniposide upregulated A20 and downregulated TNF receptor-associated factor-6 and nuclear factor kappa-B phosphorylation in the MCAO model and lipopolysaccharide-treated BV2 cells at 100 μM.

**Conclusions:**

Geniposide exhibited a neuroprotective effect via attenuating inflammatory response, as indicated by biological information analysis, *in vivo* and *in vitro* experiments, which may provide a potential direction for the application of geniposide in the treatment of ischemic stroke.

## Abbreviation

BCA: bicinchoninic acid
BP: biological process
CC: cellular component
DAMPs: damage-associated molecular patterns
ECA: external carotid artery
ELISA: enzyme-linked immunosorbent assay
GD: geniposide
GO: gene ontology
HRP: horseradish peroxidase;
IRAK: IL-1 receptor-associated kinase
IL-6: interleukin-6
iNOS: inducible nitric oxide synthase
LPS: lipopolysaccharide
MCAO: middle cerebral artery occlusion
MF: molecular function
NF-κB: nuclear factor kappa-B
PPRs: pattern recognition receptors
PVDF: polyvinylidene difluoride
rCBF: regional cerebral blood flow
RIPA: radioimmunoprecipitation assay buffer
TLRs: toll-like receptors
TRAF6: TNF receptor-associated factor-6

## 1 Introduction

Geniposide (methyl (1*S*,4*aS*,7*aS*)-1-(β-d-glucopyranosyloxy)-7-(hydroxylmethyl)-1,4*a*,5,7*a*-tetrahydrocyclopenta[*c*]pyran-4-carboxylate; C_17_H_24_O_10_) is an iridoid glucoside extracted from *Gardenia jasminoides* Ellis (Rubiaceae) fruits, a flowering plant [1]. Geniposide exhibits multiple biological properties such as antidiabetic, antioxidative, antiproliferative, and neuroprotective activities [2]. However, the main effect of geniposide in the treatment of cerebral ischemia remains unclear.

In recent years, with the rapid development of systems biology, multidirectional pharmacology, computational biology, and network pharmacology, a new discipline for systematic drug research is applied in the mechanism study [3,4]. Network pharmacology is based on the theories of systems biology, which analyzes biological systems with bioinformatics and network analysis methods and studies the mechanism of drug action from the system level. In this current study, using public data, we found that the target proteins of geniposide are mainly related to inflammation.

Neuroinflammation has been identified as an important factor in the process of secondary injury after cerebral ischemia [5,6]. Therefore, drugs or other chemical agents, which possess anti-inflammatory properties, may be effective in ameliorating ischemia-induced brain injury [7,8]. In recent years, mounting evidence has shown that geniposide could suppress neuroinflammation both *in vitro* and *in vivo* [9]. Regarding the anti-neuroinflammatory mechanisms of geniposide, recent studies have demonstrated that geniposide could inhibit the NF-κB signal pathway [10,11]. However, the nuclear translocation of NF-κB has been identified as a terminal mechanism, while the upstream mechanisms remain unclear. Thus, it is important to explore the precise molecular mechanism of geniposide.

A20 has been viewed as a negative regulator of inflammation through toll-like receptors (TLRs) and the TNF receptor pathway [12,13]. As a deubiquitinating enzyme, A20 encodes an ubiquitin-editing enzyme, which is essential for the activation of NF-κB [14]. A20 is expressed in the central nervous system, especially in microglia, as compared with the expression in other cells such as neurons, astrocytes, and brain endothelial cells [15]. A20 has been demonstrated to exert neuroprotective effects through anti-inflammatory effects in animal models of epilepsy and cerebral ischemia [16]. However, whether A20 was involved in the anti-inflammatory effect of geniposide and the terminal activation of NF-κB during cerebral ischemia remains unknown.

In the current study, we explore whether geniposide exhibited neuroprotective effect during cerebral ischemia and whether the neuroprotective effect was mediated through the A20/NF-κB signal pathway.

## 2 Materials and methods

### 2.1 Animals

Animals were housed in a humidity-controlled room on a 12 h light/dark cycle at 22°C with free access to food and water. Adult male C57BL/6 mice (8–10 weeks) were used for the experiments. Animals were assigned randomly using a random number table. All assessments were conducted by investigators who were blinded to the experimental group assignment.

A total of 150 adult male C57BL/6 mice (weighing 25–30 g) were used in our current study; 14 (9.3%) mice died before the completion of the experiment and were excluded from the study. Post-mortem examinations did not reveal the occurrence of intracerebral or subarachnoid hemorrhage in any of these animals, and no significant differences were found in the number of deaths in each group.


**Ethical statement:** All experimental procedures were approved by the committee of experimental animals of Hebei Medical University, Shijiazhuang, China (2020-AE006), and conducted in accordance with the National Institutes of Health guidelines. 

### 2.2 Reagents

Geniposide (purity >98% tested by HPLC) was purchased from Chengdu Derick Biotechnology Co. Ltd. (Sichuan, China). Lipopolysaccharide (LPS) was purchased from Sigma Chemical Co. (St. Louis, MO, USA). Antibodies against P-p65 (3033, CST), p65 (6956, CST), A20 (5630, CST), and GAPDH (2118, CST) were purchased from Cell Signaling Technology (Beverly, MA, USA). TRAF6 (38-0900, Invitrogen) was purchased from Thermo Fisher Scientific. Tumor necrosis factor-α (TNF-α; 430907, BioLegend), IL-6 (431307, BioLegend), interleukin-1β (IL-1β; 432601, BioLegend), and iNOS ELISA kits were purchased from BioLegend (CA, USA).

### 2.3 Geniposide treatment *in vivo*

Geniposide (Zelang Biotechnology Co, Nanjing, Jiangsu, China) with a purity of more than 98% was dissolved in saline. All the mice were randomly divided into five groups as follows: sham group, middle cerebral artery occlusion (MCAO) group, GD25 (25 mg/kg geniposide) group, GD75 (75 mg/kg geniposide) group, and GD150 (150 mg/kg geniposide) group. Sham and MCAO groups were treated with 0.9% normal saline only through intraperitoneal injection. Geniposide was administered through intraperitoneal injection with doses of 25, 75, or 150 mg/kg twice daily for 3 days before surgery.

### 2.4 Cerebral ischemia by MCAO

Mice were anesthetized with 1% pentobarbital (100 mg/kg, i.p.). Permanent focal cerebral ischemia was induced by right MCAO using an intraluminal filament occlusion model as previously reported [17]. First, the right carotid artery of mice was exposed to dissect the external carotid artery (ECA) and the internal carotid artery (ICA). ECA was then blocked at the level of MCA branches. Then, a filament (Guangzhou Jialing Biotech Co Ltd, Guangzhou, China) coated with silicone was inserted through the ECA and then advanced into the ICA. Finally, the filament was stopped and ligated, when a slight resistance was felt. Sham-operated mice were subjected to the same way, except for MCAO. During the operation, the body temperature of mice was maintained at 37.5 ± 0.5°C using a heating pad. Regional cerebral blood flow (rCBF) was monitored by a laser-Doppler flowmeter (moor VMS-LDF, Moor Instruments Ltd., UK) before, during, and after MCAO. The middle cerebral artery was occluded with a criterion of <25% of baseline blood flow remaining after MCAO.

### 2.5 Measurement of neurological deficits

The neurological deficits of mice were conducted before the mice were euthanized at 24 h after MCAO by an examiner blinded to the groups (*n* = 10 per group). The neurological deficits were scored using a modified scoring system reported previously [18]: 0, no deficits; 1, difficulty in fully extending the contralateral forelimb; 2, unable to extend the contralateral forelimb; 3, mild circling to the contralateral side; 4, severe circling; and 5, falling to the contralateral side.

### 2.6 Infarct volume

At 24 h after MCAO, mice (*n* = 5 per group) were anesthetized by pentobarbital, and brains were rapidly removed on the ice. Brains were sliced into five 2-mm-thick coronal sections and then stained with 2% 2,3,5-triphenyltetrazolium chloride (TTC; Sigma-Aldrich, St. Louis, MO, USA) for 10 min at 37°C. The infarct volume was calculated as follows: ([total infarct volume − {volume of intact ipsilateral hemisphere − volume of intact contralateral hemisphere}]/contralateral hemisphere volume) [19].

### 2.7 Brain edema

Brain edema was evaluated by the standard wet/dry method [20] at 24 h after MCAO (*n* = 6 per group). The two hemispheres of mice were packaged with tinfoil, respectively, to obtain wet weights on an electronic balance, and dry weights were then obtained after placing them into the oven for 24 h at 100°C. The brain water (BW) content was then calculated as follows:

BW = (wet weight − dry weight)/wet weight × 100%.

### 2.8 ELISA assay

TNF-α, IL-6, IL-1β, and iNOS levels in the brain homogenate of the ischemic hemisphere were determined using a commercial ELISA kit (NeoBioscience Technology Company, Beijing, China) at 24 h after MCAO. The brain homogenate was then centrifuged at 1,000×*g* for 15 min at 4°C to collect the supernatant. The procedure was performed following the manufacturer’s instructions. Briefly, 50 mM carbonate coating buffer (pH 9.6) was used to dissolve the antigen (10–20 μg/mL), which was then added into a 96-well enzyme label plate (100 μL/well) for overnight incubation at 4°C. Then, 150 μL of 1% bovine serum albumin was added to each well for 1 h of blocking at 37°C, and 100 μL of serum was added into each well to incubate for 2 h at 37°C. Subsequently, the serum was incubated with 100 μL of diluted horseradish peroxidase (HRP)-labeled secondary antibodies at 37°C for 1 h. Finally, the absorbance value A405 was read on a microplate reader.

### 2.9 Western blot analysis

Proteins from the mice brains and total proteins from the treated cells were extracted with the radioimmunoprecipitation assay buffer (RIPA) and were determined using the bicinchoninic acid (BCA) protein assay kit, as previously reported [21]. Equal amounts of protein (50 μg) were separated by a 10% SDS/polyacrylamide gel and then transferred onto a polyvinylidene difluoride (PVDF) membrane. The membrane was blocked with 5% BSA solution for 1 h and then incubated with primary antibodies (A20, TRAF6, and phosphorylation of NF-κB) at 4°C overnight. On the following day, the membrane was incubated with secondary antibodies. GAPDH was used as an internal control of total proteins. The densitometric values were normalized with respect to GAPDH immunoreactivity to correct for loading and transfer differences among samples. The relative density of blots was determined on an Odyssey infrared scanner (LICOR Bioscience, Lincoln, NE, USA).

### 2.10 Cell culture and treatments

BV2 cells, a microglial cell line, were cultured in Dulbecco's modified Eagle medium (DMEM) solution (Gibco, USA), supplemented with 10% fetal bovine serum (Gibco, USA) and 1% penicillin/streptomycin (Solarbio, China), in an incubator under normoxic conditions (37°C, 5% CO_2_/95% air). To test the effect of geniposide on cell viability, the cells were divided into a control group, 5 μM group (5 μM geniposide), 10 μM group (10 μM geniposide), 20 μM group (20 μM geniposide), and 100 μM group (100 μM geniposide). To explore the effect of geniposide on inflammation, LPS was administered at a concentration of 200 ng/mL. Cells were classified into different groups: control group, LPS group, and LPS + GD group (100 μM geniposide).

### 2.11 CCK-8 assay for microglial cell viability

Microglial cells were seeded into 96-well plates with the culture medium at a volume of 100 μL in each well. Cell viability was detected using the CCK-8 assay according to the manufacturer’s instructions. Briefly, 10 μL of the CCK-8 solution was added to each well. The absorbance of each well was read using a microplate reader at a test wavelength of 450 nm, with 620 nm being used as the reference wavelength.

### 2.12 Prediction of geniposide targets

SwissTargetPrediction (http://www.swisstargetprediction.ch/) is an online analysis software, which can predict the target of small molecules according to the principle of molecular similarity. We utilized this tool to predict the targets of geniposide. A protein–protein interaction (PPI) network was constructed through the STRING (v11.0, https://string-db.org/) database, which is a database of known and predicted PPIs, including direct (physical) and indirect (functional) associations.

### 2.13 Gene ontology (GO) analysis

GO analysis consists of three parts: molecular function, biological process, and cellular component. Enrichment analysis was performed via *the* David 6.8 (https://david.ncifcrf.gov/) database, which provides a comprehensive set of functional annotation tools to elaborate the biological function of the long list of genes. Briefly, to start DAVID, first click on “Functional Annotation” under “Shortcut to David tools” at the left of the home page. Then, we upload the file containing the targets of geniposide, which were predicted through SwissTargetPrediction. After the file was uploaded, we select “Functional Annotation Clustering” in the center of the page. The GO terms obtained in the drawing are arranged in descending order according to the −log10 (*P* value) of enrichment, and we used the first 30 results.

### 2.14 Statistics

All data were expressed as mean ± SEM. For statistics normally distributed, comparisons were assessed by one-way ANOVA followed by Student–Newman–Keuls tests and least significant difference (LSD) tests for multi-group comparisons and two-tailed Student’s *t*-test for two-group comparisons. For neurological deficits, Mann–Whitney *U* test was used for comparisons between the two groups. Fisher’s exact test was applied for the GO analyses. *P* < 0.05 was considered statistically significant.

## 3 Results

### 3.1 Geniposide protected against cerebral ischemic injury in mice

**Figure 1:**
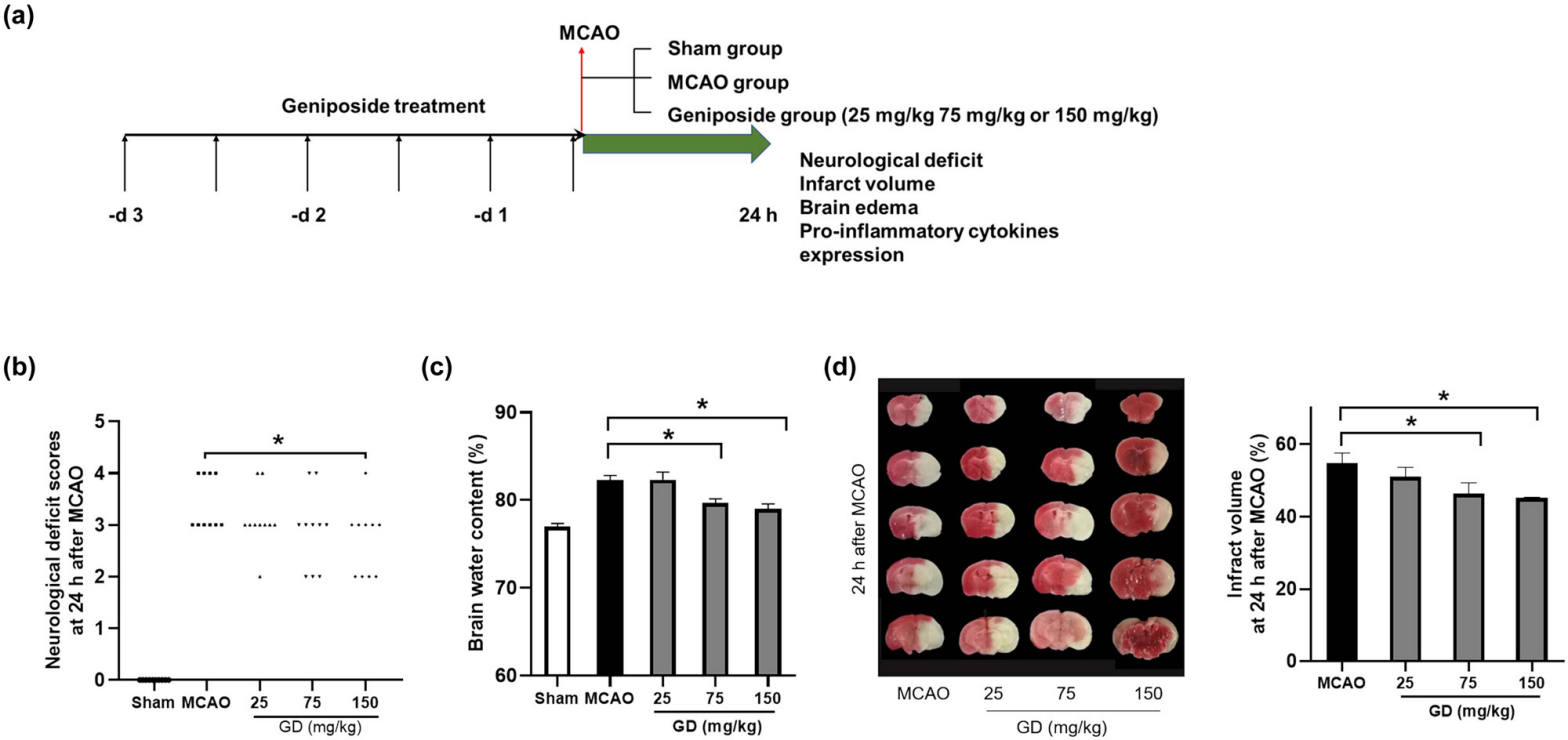
Geniposide protected against cerebral ischemia injury at 24 h after MCAO in mice. (a) Schematic diagram of the experimental design. (b) Effect of geniposide on the neurological deficit scores at 24 h after MCAO (*n* = 10 per group) (*P < 0.05, nonparametric Mann–Whitney *U* test). (c) Effect of geniposide on the brain edema at 24 h after MCAO (*n* = 6 per group) (*P < 0.05, one-way ANOVA). (d) Effect of geniposide on the infarct volume at 24 h after MCAO (*n* = 5 per group) (*P < 0.05, one-way ANOVA). Data are expressed as mean ± SEM.

The flow chart of our experiment is shown in Figure 1a. At 24 h after MCAO, mice displayed characteristics of circling to the left or even not walking spontaneously. The higher the neurological defect scores, the more serious the cerebral ischemic injury. As shown in Figure 1b, no visible neurological defect was observed in mice of the Sham group, while severe neurological defect was observed in mice of the MCAO group. The neurological defect scores in mice of the 150 mg/kg GD group were markedly reduced, compared with the MCAO group. However, there was no significant difference among the groups of MCAO, 25 mg/kg GD group, and 75 mg/kg GD group.

Brain edema was measured at 24 h after MCAO using wet/dry methods, as shown in Figure 1c. In the ischemic hemisphere, 75 and 150 mg/kg GD groups displayed ameliorated BW content (79.67 ± 0.46% vs 82.28 ± 0.53% and 79.00 ± 0.57% vs 82.28 ± 0.53%, *P* < 0.05, respectively) when compared with the MCAO group. However, no obvious difference was observed among the groups of MCAO and 25 mg/kg GD group.

Infarct volume was evaluated at 24 h after MCAO by staining, through which viable tissue is stained red and ischemic tissue is stained pale, as shown in Figure 1d. Reduced infarct volume was observed in mice of 75 and 150 mg/kg GD groups (46.28 ± 3.04% vs 54.73 ± 2.87% and 45.10 ± 0.24% vs 54.73 ± 2.87%, *P* < 0.05, respectively) when compared with the MCAO group. No obvious difference in the cerebral infarction volume was observed in the MCAO and 25 mg/kg GD group.

These results indicated that geniposide exerted a neuroprotective effect after cerebral ischemic injury. Based on the above-mentioned results, we selected 150 mg/kg GD for the following study.

### 3.2 Target prediction and construction of the PPI network of geniposide

**Figure 2:**
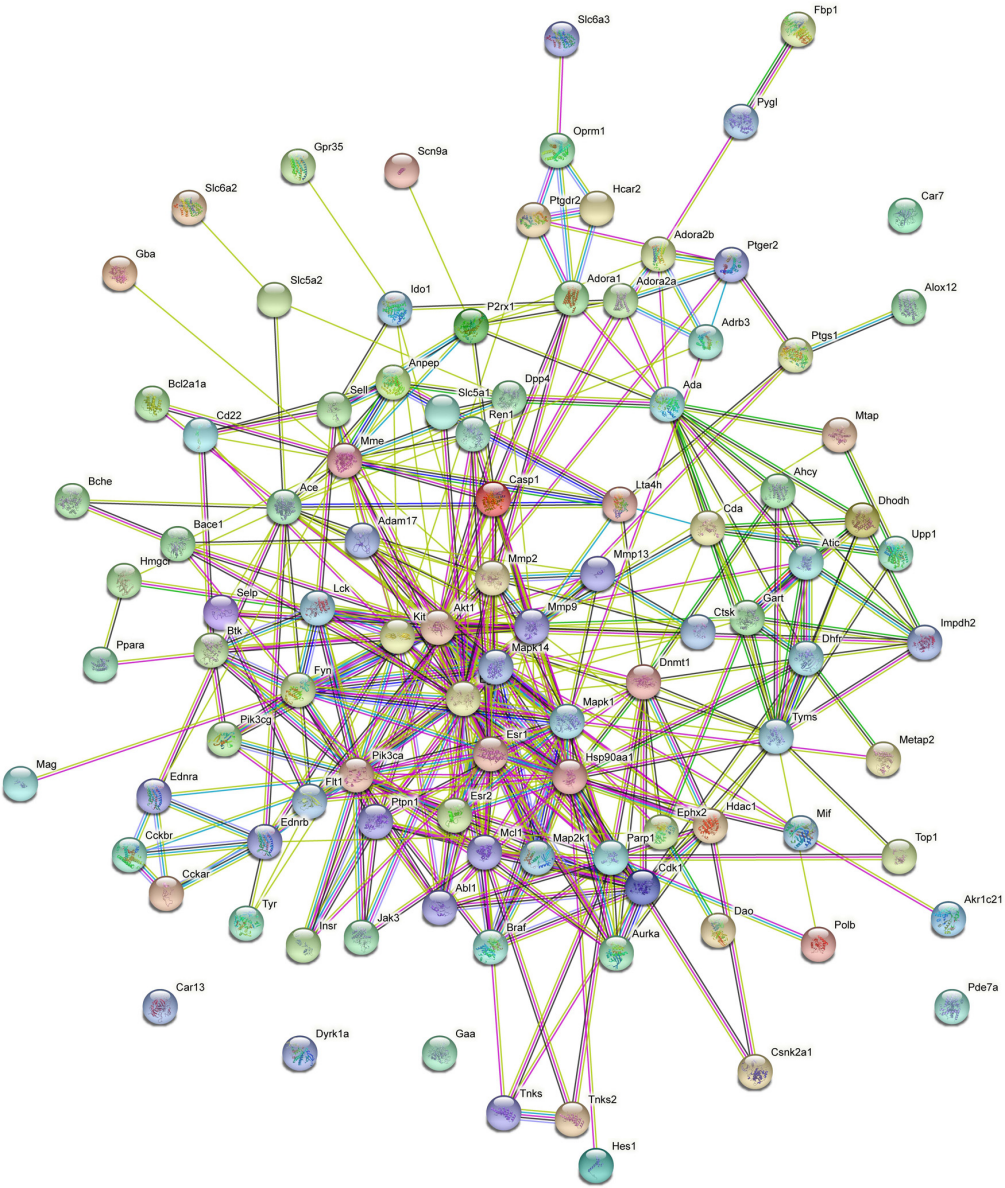
The PPI network of geniposide targets predicted by SwissTargetPrediction.

To predict the potential targets of geniposide, we queried geniposide targets using the SwissTargetPrediction tool. One hundred geniposide targets were predicted with SwissTargetPrediction (Supplemental materials). To construct an interaction network, we studied the PPI relationship via the STRING database. This network consisted of the above 100 terms, including 100 nodes and 400 edges (Figure 2). As shown in Figure 2, the interactions mainly exist in inflammatory-related proteins, such as MAPK, MMP9, Akt1, ESR1, HSP90, and so on, which suggested that the neuroprotective effect of geniposide may be mainly attributed to its anti-inflammatory effect.

### 3.3 GO analyses of geniposide

**Figure 3:**
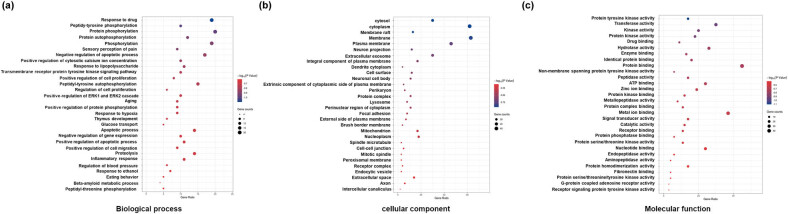
GO pathway enrichment of geniposide targets predicted by SwissTargetPrediction. GO analysis included three parts: the biological process (a), cellular component (b), and molecular function (c), which, respectively, describe the biological processes involved, the cellular environments in which they are located, and the molecular functions of potential gene products. Enrichment analysis was performed via the DAVID 6.8 database. The enriched terms of GO analysis are arranged in descending order according to −log10 (*P* value). BP: biological process; MF: molecular function; CC: cellular component.

To investigate the biological function and potential mechanism of geniposide, GO analyses were performed via the DAVID database. Biological process analysis was mainly enriched in terms of inflammatory response and negative regulation of apoptotic progress. Molecular function analysis was primarily enriched in terms of protein binding (Figure 3). These findings suggested that geniposide participated in biological functions that were closely related to the inflammatory response.

### 3.4 Geniposide downregulated the expression of inflammatory cytokines

**Figure 4:**
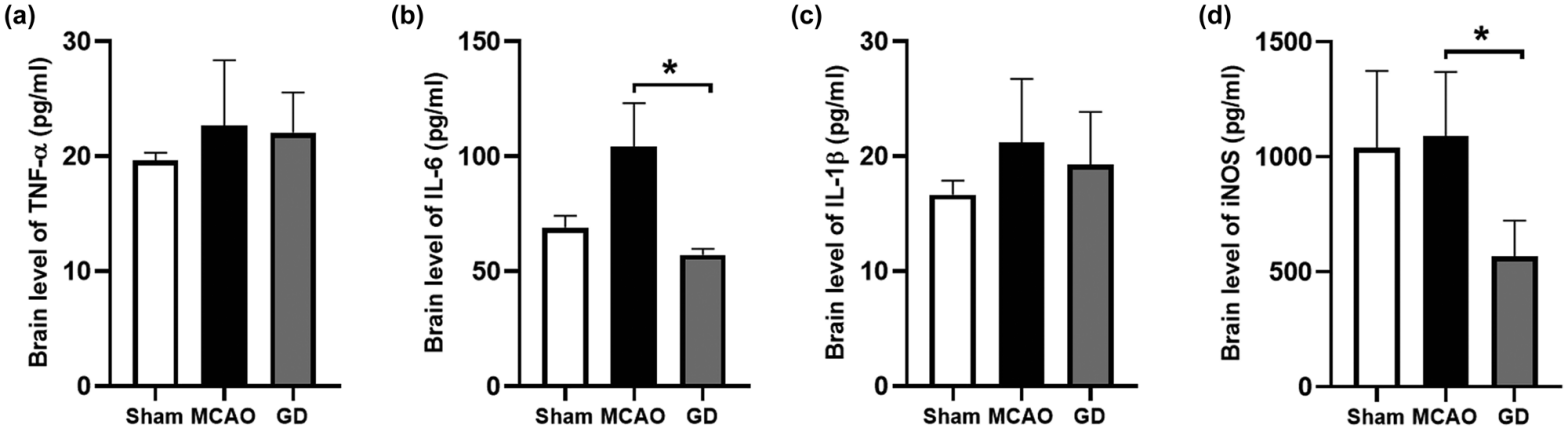
Geniposide suppressed the overproduction of pro-inflammatory cytokines at 24 h after MCAO in mice. The expression of TNF-α (a), IL-6 (b), IL-1β (c), and iNOS (d) in the penumbra of mice brains was detected by ELISA (*n* = 4–5 per group) (*P < 0.05, one-way ANOVA).

To confirm the accuracy of target prediction and GO analysis, we thus investigated the expression of pro-inflammatory cytokines in the brain at 24 h after cerebral ischemia. The results showed that the expression of TNF-α, IL-6, IL-1β, and iNOS was upregulated after MCAO (Figure 4). Importantly, after geniposide treatment, the expression of IL-6 and iNOS was significantly reduced, which suggested that geniposide exhibited an anti-inflammatory effect after cerebral ischemia.

### 3.5 Geniposide upregulated the expression of A20 and inhibited the activation of NF-κB

**Figure 5:**
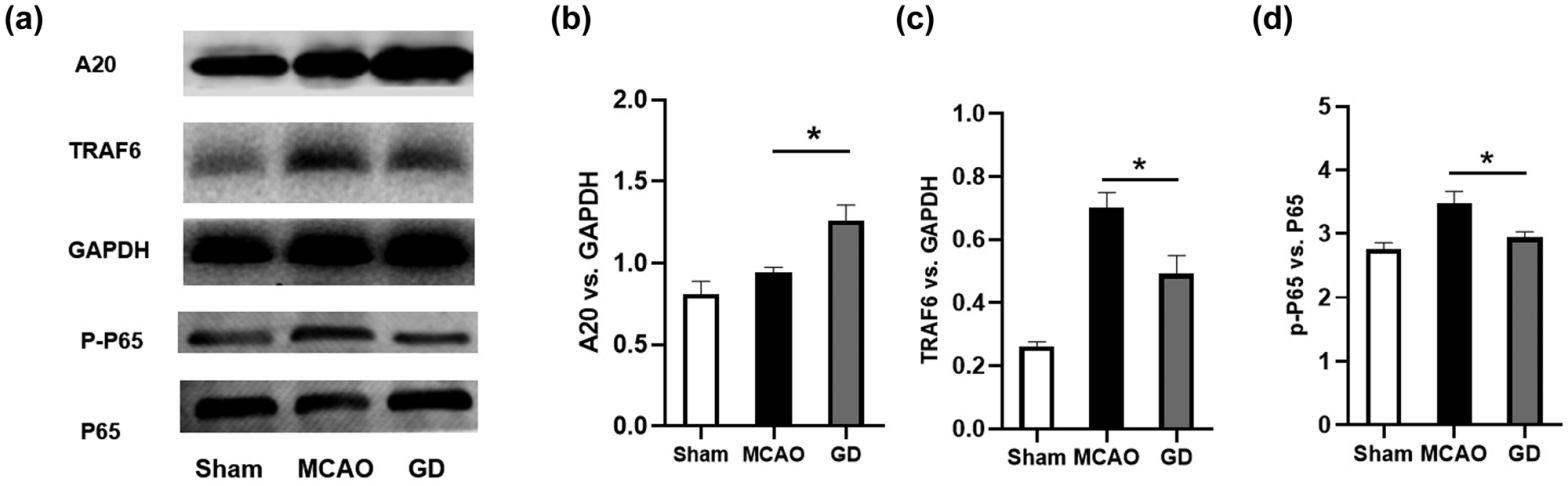
Geniposide inhibited inflammation by upregulating A20 expression and downregulating TRAF6 expression and NF-κB phosphorylation. The expression of A20, TRAF6, p-NF-κB 65, and p-NF-κB in the penumbra of mice brains was detected by western blot (a). Quantification of the expression of A20 (b), TRAF6 (c), and phosphorylation of NF-κB (d) by Image-pro-plus (*n* = 4 per group) (*P < 0.05, one-way ANOVA).

We further explored the expression of A20, TRAF6, and the phosphorylation of NF-κB, which was the upstream signal of IL-6 and iNOS, in the penumbra of the brain tissue. As shown in Figure 5, the expression of A20, TRAF6, and the phosphorylation of NF-κB was increased after MCAO. Importantly, geniposide ameliorated the ischemia-induced upregulation of TRAF-6 and p-NF-κB significantly. Besides, geniposide significantly increased the expression of A20 when compared with the MCAO group.

As IL-6 and iNOS were mainly produced by activated microglia, we used microglia cell lines, BV2 cells, to examine the inhibitory effect of geniposide on microglia activation and its underlying mechanisms.

**Figure 6:**
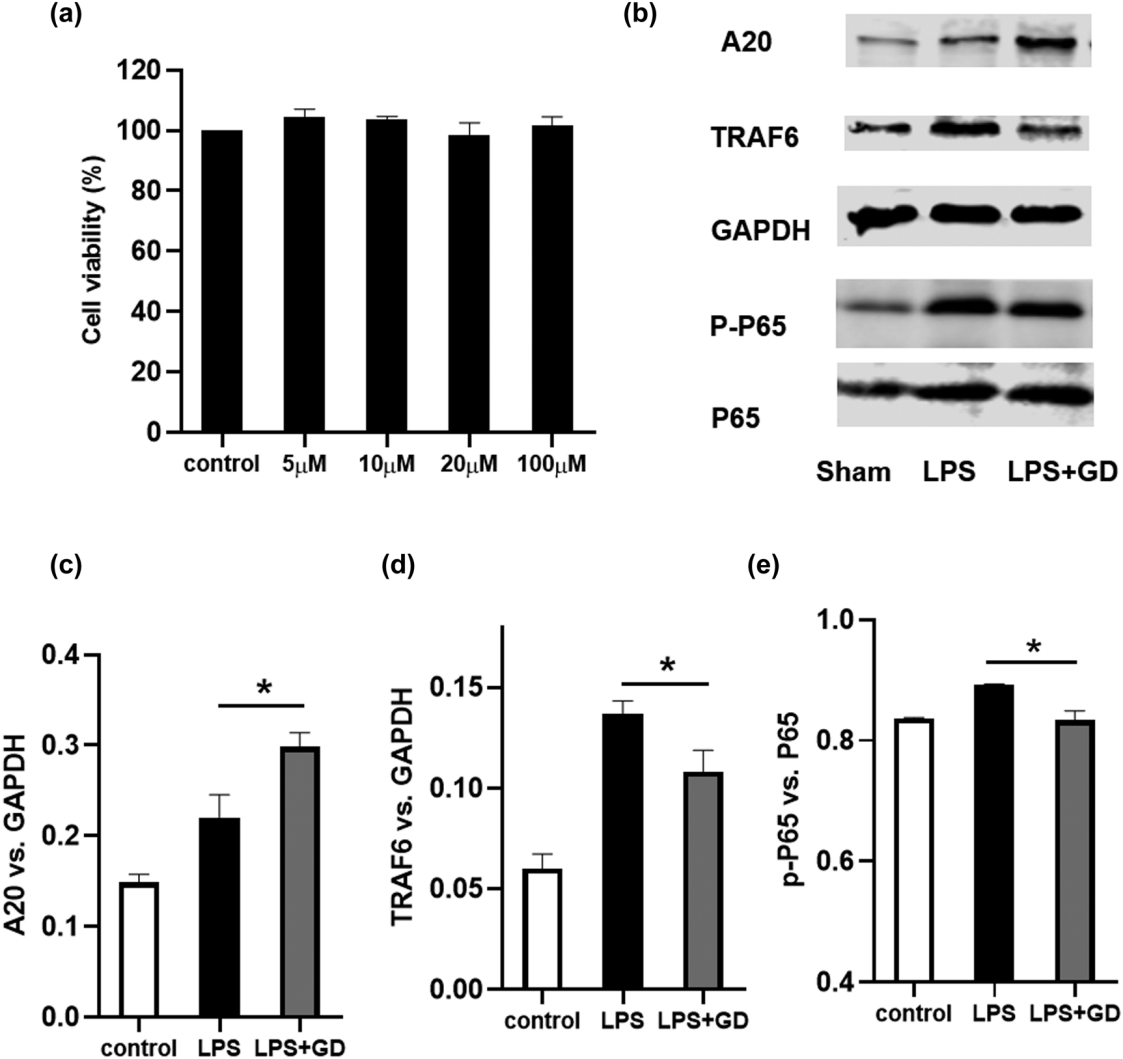
Geniposide inhibited LPS-induced inflammation by upregulating A20 expression and downregulating TRAF6 expression and NF-κB phosphorylation in BV2 cells. The cell viability was examined by CCK8 assay (a). The expression of A20, TRAF6, p-NF-κB 65, and p-NF-κB in BV2 cells was detected by western blot (b). Quantification of the expression of A20 (c), TRAF6 (d), and phosphorylation of NF-κB (e) by Image-pro-plus. The results shown are representative of three independent experiments (*P < 0.05, one-way ANOVA). Data are expressed as mean ± SEM; *n* = 4–6 per group.

To evaluate whether geniposide affected the cell viability of BV2 cells, the cell viability was assessed by CCK8 assay. As shown in Figure 6a, geniposide exerted no significant toxic effects on BV2 at four concentrations (5, 10, 20, and 100 μM) after 24 h treatment. We then selected 100 μM in subsequent studies.

LPS (200 ng/mL) was then used to activate microglia. As shown in Figure 6b–e, the expression of A20, TRAF6, and the phosphorylation of NF-κβ was increased after LPS treatment. Importantly, geniposide ameliorated the LPS-induced upregulation of TRAF-6 and p-NF-κβ significantly. Moreover, geniposide significantly increased the expression of A20 when compared with the LPS group.

## 4 Discussion

In the current study, we first investigated the neuroprotective role of geniposide during cerebral ischemia in mice and then explored the potential targets of geniposide using bioinformatics tools, including SwissTargetPrediction and analyses of the GO pathway and PPI network. With the bioinformatics tools, we identified that the protective effect of geniposide may be mediated through its anti-inflammatory properties. We then verified the anti-inflammatory effect of geniposide and revealed that A20 may serve as a novel target in the treatment of cerebral ischemia.

Geniposide is found in 40 species belonging to various families, especially Rubiaceae [22]. In the 2015 edition, geniposide has been listed as the quantitative component for quality evaluation of nearly 20 Chinese Patent Medicine preparations containing *G. jasminoides* fruits, such as the Bazheng mixture, Longdan Xiegan pills, Qingkailing soft capsules, Niuhuang Shangqing soft capsules, and Zhizi Jinhua pills (National Pharmacopoeia Commission, 2020). Multiple lines of pharmacological evidence have demonstrated the various biological properties of geniposide, such as anti-inflammatory [23], antioxidative [24], antidiabetic [25], and neuroprotective [26]. In a LPS-induced mouse model of mastitis, geniposide (2.5, 5, and 10 mg/kg *in vivo*; 25, 50, and 100 µg/mL *in vitro*) has been demonstrated to alleviate inflammatory response by the inhibition of TLR4. Moreover, evidence has shown that geniposide may serve as a potential drug to prevent the early progression of AD [9]. Geniposide (i.g., 25 and 50 mg/kg) was demonstrated to inhibit inflammation and improve barrier dysfunction by activating the AMPK pathway. Additionally, geniposide treatment could protect against the above dysfunction by attenuating axonal mitochondrial fragmentation, trafficking impairments, and reactive oxygen species (ROS) elevation, protecting the synaptic loss, abnormal spine density and morphology, and ameliorating the decrease in synapse-related proteins. The neuroprotective effect of geniposide in cerebral ischemia has been investigated in previous studies, which used transient MCAO rat models. However, the transient MCAO model, which mimics stroke with reperfusion, does not accurately reflect the majority of clinical stroke cases. Therefore, the argument for studying permanent MCAO as a primary model is made and supported [27]. Currently, the effect of geniposide has not been investigated in a permanent MCAO model. Thus, it is essential to investigate the neuroprotective effect of geniposide in a permanent MCAO model; our results showed that geniposide exerted a neuroprotective effect at 24 h after permanent MCAO, as indicated by improved neurofunction, as well as reduced brain edema and infarct volume. However, the specific targets of geniposide remained unclear.

Through the SwissTargetPrediction database, we verified 100 geniposide targets, which were enriched in the pathway of inflammatory and cell apoptosis as indicated by GO analysis. Accumulated evidence has demonstrated that inflammation plays a crucial role after cerebral ischemia [8]. After cerebral ischemia, the ischemic tissue was divided into two areas, infarct core and penumbra. Excessive inflammatory response occurred in the penumbra, which may further aggravate the infarct volume [28]. Considering the important role of inflammation in acute cerebral ischemia and to confirm the anti-inflammatory properties of geniposide, we examined the expression of pro-inflammatory cytokines. Our results demonstrated that geniposide significantly reduced the expression of pro-inflammatory factors, such as IL-6 and iNOS.

Microglia, identified as the macrophage of the central nervous system, play important roles in neuroinflammation after cerebral ischemia [29]. After cerebral ischemia, the dying neurons release damage-associated molecular patterns (DAMPs), which bind to the pattern recognition receptors (PPRs) in the membrane of microglia, leading to the activation of microglia and the overproduction of pro-inflammatory cytokines, such as IL-6 and iNOS [30]. The pro-inflammatory cytokines further aggravate ischemia-induced damage, resulting in severe neurological deficits [5]. During cerebral ischemia, the overproduction of pro-inflammatory cytokines, such as IL-6 and iNOS, was mediated by NF-κB. Evidence has shown that IL-1 receptor-associated kinase (IRAK) is activated by phosphorylation and then associates with TRAF6, leading to the activation of downstream NF-κB signaling pathways [5]. The relationship between TRAF6 and NF-κB has been elaborated in accumulated studies. TRAF6 is an important E3 ubiquitin ligase, which is crucial in mediating the activation of NF-κB [31]. Geniposide has been demonstrated to ameliorate the alterations of TLR4/MyD88 in diabetic mice and SH-SY5Y cells [32]. Geniposide also regulated NF-κB activation by reacting with the free sulfhydryl groups of cysteine. Therefore, we wanted to know whether the anti-inflammatory effect of geniposide was associated with the TRAF6/NF-κB signaling pathway in our mouse model of MCAO. In our present study, we found that geniposide inhibited the expression of TRAF6 and the activation of NF-κB after cerebral ischemia.

**Figure 7:**
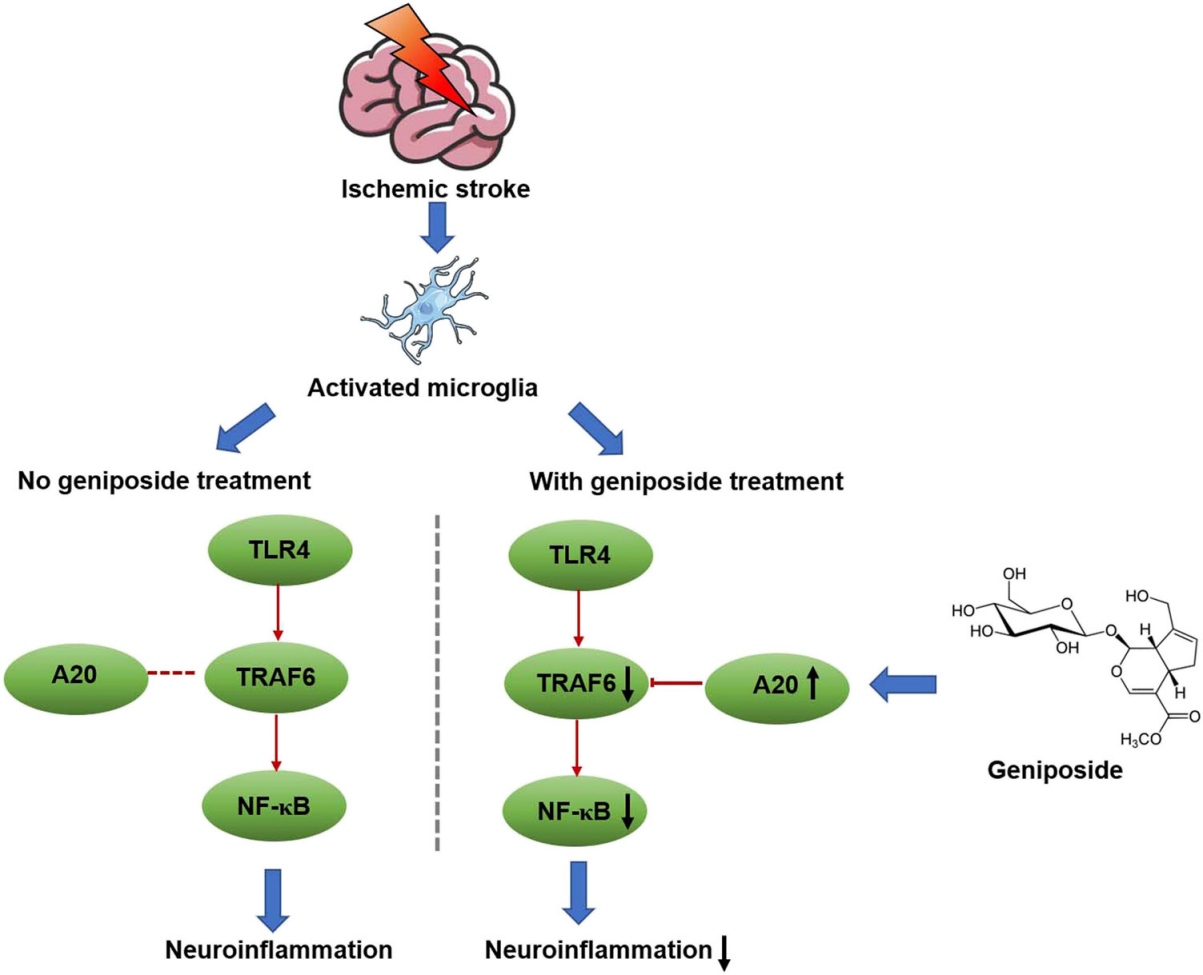
Mechanisms involved in the anti-neuroinflammatory effects of geniposide.

Importantly, we demonstrated that geniposide could upregulate the expression of A20 after cerebral ischemia, which was not found as a geniposide target via SwissTargetPrediction but was predicted with the highest score in GEO datasets of cerebral ischemia, suggesting that A20 was a potential geniposide target. A20, a zinc finger protein, functions as a potent negative regulator of the inflammatory response and feedback inhibitor of NF-κB. Mounting evidence has demonstrated that A20 could regulate the activity of TRAF6 by direct K63-linked deubiquitination and by K48-linked ubiquitination via the ubiquitin–proteasome system. These results suggested that geniposide inhibited the expression of TRAF6 and the phosphorylation of NF-κB, at least partly, by increasing the expression of A20 (Figure 7).

## 5 Conclusions

Our current study suggested that upregulation of A20 was involved in the neuroprotective and anti-inflammatory effect of geniposide during cerebral ischemia. The neuroprotective effect of geniposide was, at least partly, through the A20/NF-κB pathway.

## Supplementary Material

Supplementary Table 1

Supplementary Table 2
