# Supplementary material for: Geniposide protected against cerebral ischemic injury through the anti-inflammatory effect via the NF-κB signaling pathway
Source: Transl Neurosci. 2023 Jun 9;14(1):20220273. doi: 10.1515/tnsci-2022-0273 (PMC10276575; doi:10.1515/tnsci-2022-0273)
Supplement: Supplementary Table 1 [file tnsci-2022-0273-sm1.pdf]

Target	Common name
Adenosine A1 receptor (by homology)	Adora1
Adenosine A2a receptor (by homology)	Adora2a
Adenosine deaminase (by homology)	Ada
Sodium/glucose cotransporter 2 (by homology)	Slc5a2
Beta-glucocerebrosidase (by homology)	Gba
Adenosine A2b receptor (by homology)	Adora2b
Carbonic anhydrase XIII (by homology)	Ca13
Tyrosinase (by homology)	Tyr
Carbonic anhydrase VII (by homology)	Ca7
Matrix metalloproteinase-2 (by homology)	Mmp2
SGLT1 protein (by homology)	Slc5a1
Epoxide hydratase (by homology)	Ephx2
Aldo-keto reductase family 1 member C21 (by homology)	Akr1c21
Myelin-associated glycoprotein	Mag
AICAR transformylase (by homology)	Atic
Arachidonate 12-lipoxygenase (by homology)	Alox12
Collagenase 3 (by homology)	Mmp13
Liver glycogen phosphorylase (by homology)	Pygl
Epidermal growth factor receptor erbB1 (by homology)	Egfr
Heat shock protein HSP 90-alpha (by homology)	Hsp90aa1
Endothelin receptor ET-A (by homology)	Ednra
Methionine aminopeptidase 2 (by homology)	Metap2
Matrix metalloproteinase 9 (by homology)	Mmp9
Tyrosine-protein kinase ABL (by homology)	Abl1
Norepinephrine transporter (by homology)	Slc6a2
Tyrosine-protein kinase LCK (by homology)	Lck
Serine/threonine-protein kinase Aurora-A (by homology)	Aurka
Tyrosine-protein kinase BTK (by homology)	Btk
Inosine-5'-monophosphate dehydrogenase 2 (by homology)	Impdh2
ADAM17 (by homology)	Adam17
Neprilysin (by homology)	Mme
DNA topoisomerase I (by homology)	Top1
Angiotensin-converting enzyme (by homology)	Ace
Induced myeloid leukemia cell differentiation protein Mcl-1 ho	Mcl1
Bcl-2-related protein A1 (by homology)	Bcl2a1
Lysosomal alpha-glucosidase (by homology)	Gaa
Cytidine deaminase	Cda
G protein-coupled receptor 44 (by homology)	Ptgdr2
D-amino-acid oxidase (by homology)	Dao
Tyrosine-protein kinase JAK3 (by homology)	Jak3
Dual specificity mitogen-activated protein kinase kinase 1 (by	Map2k1
P-selectin (by homology)	Selp
GAR transformylase	Gart
MAP kinase ERK2 (by homology)	Mapk1
Estrogen receptor alpha (by homology)	Esr1
Estrogen receptor beta (by homology)	Esr2
Aminopeptidase N (by homology)	Anpep
DNA (cytosine-5)-methyltransferase 1 (by homology)	Dnmt1
Leukotriene A4 hydrolase (by homology)	Lta4h
Adenosylhomocysteinase (by homology)	Ahcy
MAP kinase p38 alpha (by homology)	Mapk14
Leukocyte adhesion molecule-1 (by homology)	Sell
High affinity cAMP-specific 3',5'-cyclic phosphodiesterase 7A	Pde7a
Dipeptidyl peptidase IV (by homology)	Dpp4
Histone deacetylase 1 (by homology)	Hdac1
Dihydroorotate dehydrogenase (by homology)	Dhodh
Hydroxycarboxylic acid receptor 2 (by homology)	Hcar2

HMG-CoA reductase (by homology)	Hmgcr
Renin (by homology)	Ren1
Insulin receptor	Insr
Thymidylate synthase (by homology)	Tyms
S-methyl-5-thioadenosine phosphorylase	Mtap
Indoleamine 2,3-dioxygenase 1 (by homology)	Ido1
Poly [ADP-ribose] polymerase-1	Parp1
Mu opioid receptor (by homology)	Oprm1
Endothelin receptor ET-B (by homology)	Ednrb
Beta-secretase 1 (by homology)	Bace1
PI3-kinase p110-alpha subunit (by homology)	Pik3ca
Caspase-1 (by homology)	Casp1
Vascular endothelial growth factor receptor 1 (by homology)	Flt1
Mast/stem cell growth factor receptor Kit (by homology)	Kit
Macrophage migration inhibitory factor (by homology)	Mif
Phosphatidylinositol 4,5-bisphosphate 3-kinase catalytic subun	Pik3cg
Cholecystokinin A receptor (by homology)	Cckar
Cholecystokinin B receptor (by homology)	Cckbr
Protein-tyrosine phosphatase 1B (by homology)	Ptpn1
Dopamine transporter (by homology)	Slc6a3
Transcription factor HES-1	Hes1
Fructose-1,6-bisphosphatase 1 (by homology)	Fbp1
Uridine phosphorylase 1	Upp1
P2X purinoceptor 1	P2rx1
Tankyrase-2 (by homology)	Tnks2
Tankyrase-1 (by homology)	Tnks
Cyclooxygenase-1 (by homology)	Ptgs1
Beta-3 adrenergic receptor (by homology)	Adrb3
Sodium channel protein type 9 subunit alpha (by homology)	Scn9a
Cathepsin K (by homology)	Ctsk
Cyclin-dependent kinase 1 (by homology)	Cdk1
Dual-specificity tyrosine-phosphorylation regulated kinase 1A	Dyrk1a
Peroxisome proliferator-activated receptor alpha (by homolog	Ppara
RAC-alpha serine/threonine-protein kinase (by homology)	Akt1
Prostanoid EP2 receptor (by homology)	Ptger2
DNA polymerase beta (by homology)	Polb
G-protein coupled receptor 35	Gpr35
Casein kinase II alpha (by homology)	Csnk2a1
Dihydrofolate reductase (by homology)	Dhfr
Butyrylcholinesterase (by homology)	Bche
Serine/threonine-protein kinase B-raf (by homology)	Braf
B-cell receptor CD22	Cd22
Tyrosine-protein kinase FYN (by homology)	Fyn

Uniprot ID	ChEMBL ID	Target Class	Probability*
Q60612	CHEMBL3688	Family A G protein-coupled recepto	0.113286
Q60613	CHEMBL2115	Family A G protein-coupled recepto	0.113286
P03958	CHEMBL3206	Hydrolase	0.113286
Q923I7	CHEMBL107530	Electrochemical transporter	0.113286
P17439	CHEMBL2278	Enzyme	0.113286
Q60614	CHEMBL2237	Family A G protein-coupled recepto	0.113286
Q9D6N1	CHEMBL2186	Enzyme	0.113286
P11344	CHEMBL5346	Oxidoreductase	0.113286
Q9ERQ8	CHEMBL2216	Enzyme	0.113286
P33434	CHEMBL3095	Protease	0.113286
Q9QXI6	CHEMBL174452	Unclassified protein	0.113286
P34914	CHEMBL4140	Protease	0.113286
Q91WR5	CHEMBL107527	Enzyme	0.113286
P20917	CHEMBL125041	Unclassified protein	0.113286
Q9CWJ9	CHEMBL2277	Enzyme	0
P39655	CHEMBL3225	Enzyme	0
P33435	CHEMBL363835	Hydrolase	0
Q9ET01	CHEMBL3008	Enzyme	0
Q01279	CHEMBL3608	Kinase	0
P07901	CHEMBL4197	Other cytosolic protein	0
Q61614	CHEMBL2286	Family A G protein-coupled recepto	0
O08663	CHEMBL107527	Protease	0
P41245	CHEMBL2214	Protease	0
P00520	CHEMBL3099	Kinase	0
O55192	CHEMBL2370	Electrochemical transporter	0
P06240	CHEMBL2480	Kinase	0
P97477	CHEMBL2211	Kinase	0
P35991	CHEMBL325947	Kinase	0
P24547	CHEMBL3169	Oxidoreductase	0
Q9Z0F8	CHEMBL4379	Protease	0
Q61391	CHEMBL2642	Protease	0
Q04750	CHEMBL2814	Isomerase	0
P09470	CHEMBL2994	Protease	0
P97287	CHEMBL5768	Unclassified protein	0
Q07440	CHEMBL129323	Unclassified protein	0
P70699	CHEMBL166766	Hydrolase	0
P56389	CHEMBL2110	Enzyme	0
Q9Z2J6	CHEMBL2291	Family A G protein-coupled recepto	0
P18894	CHEMBL233106	Enzyme	0
Q62137	CHEMBL5250	Kinase	0
P31938	CHEMBL5860	Enzyme	0
Q01102	CHEMBL2455	Adhesion	0
Q64737	CHEMBL3690	Ligase	0
P63085	CHEMBL2207	Kinase	0
P19785	CHEMBL3065	Nuclear receptor	0
O08537	CHEMBL2995	Nuclear receptor	0
P97449	CHEMBL218914	Protease	0
P13864	CHEMBL335119	Writer	0
P24527	CHEMBL3738	Protease	0
P50247	CHEMBL2389	Enzyme	0
P47811	CHEMBL2336	Kinase	0
P18337	CHEMBL3162	Adhesion	0
P70453	CHEMBL204070	Phosphodiesterase	0
P28843	CHEMBL3883	Protease	0
O09106	CHEMBL4001	Eraser	0
O35435	CHEMBL2991	Oxidoreductase	0
Q9EP66	CHEMBL4420	Family A G protein-coupled recepto	0

Q01237	CHEMBL2764	Oxidoreductase	0
P06281	CHEMBL2615	Protease	0
P15208	CHEMBL3187	Kinase	0
P07607	CHEMBL3160	Transferase	0
Q9CQ65	CHEMBL2663	Enzyme	0
P28776	CHEMBL107529	Enzyme	0
P11103	CHEMBL3740	Enzyme	0
P42866	CHEMBL2858	Family A G protein-coupled recepto	0
P48302	CHEMBL168161	Family A G protein-coupled recepto	0
P56818	CHEMBL4593	Protease	0
P42337	CHEMBL2499	Enzyme	0
P29452	CHEMBL4800	Protease	0
P35969	CHEMBL3516	Kinase	0
P05532	CHEMBL203479	Kinase	0
P34884	CHEMBL192649	Enzyme	0
Q9JHG7	CHEMBL218915	Enzyme	0
O08786	CHEMBL2798	Family A G protein-coupled recepto	0
P56481	CHEMBL2854	Family A G protein-coupled recepto	0
P35821	CHEMBL3336	Phosphatase	0
Q61327	CHEMBL2799	Electrochemical transporter	0
P35428	CHEMBL107529	Transcription factor	0
Q9QXD6	CHEMBL5360	Enzyme	0
P52624	CHEMBL3718	Enzyme	0
P51576	CHEMBL5496	Ligand-gated ion channel	0
Q3UES3	CHEMBL323270	Enzyme	0
Q6PFX9	CHEMBL323270	Enzyme	0
P22437	CHEMBL2649	Enzyme	0
P25962	CHEMBL4030	Family A G protein-coupled recepto	0
Q62205	CHEMBL341441	Voltage-gated ion channel	0
P55097	CHEMBL107527	Protease	0
P11440	CHEMBL4084	Kinase	0
Q61214	CHEMBL4750	Kinase	0
P23204	CHEMBL2128	Nuclear receptor	0
P31750	CHEMBL5859	Kinase	0
Q62053	CHEMBL2488	Family A G protein-coupled recepto	0
Q8K409	CHEMBL4565	Enzyme	0
Q9ES90	CHEMBL239081	Family A G protein-coupled recepto	0
Q60737	CHEMBL3537	Kinase	0
P00375	CHEMBL4564	Oxidoreductase	0
Q03311	CHEMBL2528	Hydrolase	0
P28028	CHEMBL233106	Kinase	0
P35329	CHEMBL107527	Adhesion	0
P39688	CHEMBL4517	Kinase	0
