# Supplementary material for: Geniposide protected against cerebral ischemic injury through the anti-inflammatory effect via the NF-κB signaling pathway
Source: Transl Neurosci. 2023 Jun 9;14(1):20220273. doi: 10.1515/tnsci-2022-0273 (PMC10276575; doi:10.1515/tnsci-2022-0273)
Supplement: Supplementary Table 2 [file tnsci-2022-0273-sm2.pdf]

Category	Term	Count	Term	ratio	P value	PValue	Genes	List Total
GOTERM_	GO:004249	19	response t	19	12.47953	3.31E-13	14360, 131	100
GOTERM_	GO:001810	10	peptidyl-ty	10	10.12084	7.57E-11	14360, 168	100
GOTERM_	GO:000646	20	protein ph	20	9.567507	2.71E-10	14360, 142	100
GOTERM_	GO:004677	12	protein au	12	8.308698	4.91E-09	14360, 168	100
GOTERM_	GO:001631	19	phosphory	19	8.285688	5.18E-09	14360, 142	100
GOTERM_	GO:001923	9	sensory pe	9	8.029563	9.34E-09	13617, 136	100
GOTERM_	GO:004306	17	negative re	17	7.152972	7.03E-08	11491, 173	100
GOTERM_	GO:000720	10	positive re	10	6.922633	1.19E-07	13617, 136	100
GOTERM_	GO:003249	11	response t	11	6.899809	1.26E-07	13617, 136	100
GOTERM_	GO:007037	10	positive re	10	6.042344	9.07E-07	13617, 263	100
GOTERM_	GO:000716	8	transmemb	8	5.90074	1.26E-06	14360, 168	100
GOTERM_	GO:000828	15	positive re	15	5.83411	1.47E-06	11491, 433	100
GOTERM_	GO:003808	6	peptidyl-ty	6	5.545455	2.85E-06	14360, 168	100
GOTERM_	GO:004212	10	regulation	10	5.366931	4.30E-06	14360, 168	100
GOTERM_	GO:000756	9	aging	9	5.31193	4.88E-06	13618, 131	100
GOTERM_	GO:000193	9	positive re	9	5.114821	7.68E-06	13617, 136	100
GOTERM_	GO:000166	9	response t	9	4.979677	1.05E-05	13617, 115	100
GOTERM_	GO:004853	6	thymus de	6	4.905058	1.24E-05	26395, 221	100
GOTERM_	GO:001575	5	glucose tra	5	4.870681	1.35E-05	13617, 246	100
GOTERM_	GO:000691	14	apoptotic	14	4.863652	1.37E-05	12362, 120	100
GOTERM_	GO:001062	10	negative re	10	4.825969	1.49E-05	14360, 263	100
GOTERM_	GO:004306	11	positive re	11	4.821632	1.51E-05	12362, 173	100
GOTERM_	GO:003033	9	positive re	9	4.803979	1.57E-05	26395, 136	100
GOTERM_	GO:000650	14	proteolysis	14	4.768183	1.71E-05	16790, 123	100
GOTERM_	GO:000695	11	inflammato	11	4.722524	1.89E-05	19224, 203	100
GOTERM_	GO:000821	6	regulation	6	4.648656	2.25E-05	13617, 136	100
GOTERM_	GO:004547	7	response t	7	4.353654	4.43E-05	14360, 131	100
GOTERM_	GO:004275	5	eating beh	5	4.266864	5.41E-05	11540, 114	100
GOTERM_	GO:005043	4	beta-amyl	4	4.072346	8.47E-05	17380, 114	100
GOTERM_	GO:001810	5	peptidyl-th	5	4.052763	8.86E-05	21951, 116	100
GOTERM_	GO:190435	3	negative re	3	4.052043	8.87E-05	21951, 744	100
GOTERM_	GO:000697	7	response t	7	4.043287	9.05E-05	19224, 263	100
GOTERM_	GO:004542	5	positive re	5	3.974581	1.06E-04	15519, 136	100
GOTERM_	GO:004231	4	vasoconstr	4	3.839393	1.45E-04	13617, 136	100
GOTERM_	GO:004665	3	tetrahydro	3	3.752568	1.77E-04	13361, 144	100
GOTERM_	GO:004340	5	positive re	5	3.664088	2.17E-04	13649, 142	100
GOTERM_	GO:004317	4	peptide ca	4	3.643375	2.27E-04	16790, 169	100
GOTERM_	GO:190018	4	positive re	4	3.584403	2.60E-04	14360, 116	100
GOTERM_	GO:003057	4	collagen ca	4	3.528136	2.96E-04	17395, 173	100
GOTERM_	GO:001062	10	positive re	10	3.467476	3.41E-04	16818, 263	100
GOTERM_	GO:000716	18	signal tran	18	3.360432	4.36E-04	18583, 109	100
GOTERM_	GO:000182	3	histamine	3	3.357737	4.39E-04	13617, 114	100
GOTERM_	GO:003222	3	negative re	3	3.357737	4.39E-04	13618, 173	100
GOTERM_	GO:001003	5	response t	5	3.353481	4.43E-04	12229, 144	100
GOTERM_	GO:003165	3	positive re	3	3.213163	6.12E-04	13649, 114	100
GOTERM_	GO:000673	4	one-carbo	4	3.193682	6.40E-04	11615, 133	100
GOTERM_	GO:004362	5	response t	5	3.117491	7.63E-04	15519, 173	100
GOTERM_	GO:004632	4	positive re	4	3.112981	7.71E-04	16337, 173	100
GOTERM_	GO:004298	7	regulation	7	3.102282	7.90E-04	14360, 168	100
GOTERM_	GO:000197	3	adenosine	3	3.089777	8.13E-04	11539, 115	100
GOTERM_	GO:001810	6	peptidyl-s	6	3.085455	8.21E-04	21951, 116	100
GOTERM_	GO:007126	5	cellular res	5	3.053414	8.84E-04	13617, 171	100
GOTERM_	GO:004590	4	positive re	4	2.932321	0.001169	13649, 139	100
GOTERM_	GO:000963	5	response t	5	2.89508	0.001273	22171, 202	100
GOTERM_	GO:004867	3	regulation	3	2.886826	0.001298	26395, 114	100
GOTERM_	GO:006002	3	Bergmann	3	2.886826	0.001298	26395, 113	100
GOTERM_	GO:005193	4	regulation	4	2.835857	0.001459	13618, 115	100

GOTERM_	GO:00303	5	positive re	5	2.821954	0.001507	13649, 11	100
GOTERM_	GO:00459	4	positive re	4	2.718501	0.001912	19224, 13	100
GOTERM_	GO:00425	3	positive re	3	2.587198	0.002587	14360, 16	100
GOTERM_	GO:00071	4	adenylate	4	2.586805	0.002589	11540, 11	100
GOTERM_	GO:00515	4	response t	4	2.562191	0.00274	13162, 221	100
GOTERM_	GO:00076	5	circadian r	5	2.532785	0.002932	13649, 221	100
GOTERM_	GO:00075	7	heart deve	7	2.505182	0.003125	13617, 26	100
GOTERM_	GO:00457	3	positive re	3	2.470154	0.003387	17395, 13	100
GOTERM_	GO:00322	3	positive re	3	2.470154	0.003387	11540, 12	100
GOTERM_	GO:19043	3	positive re	3	2.470154	0.003387	21951, 26	100
GOTERM_	GO:19012	4	negative r	4	2.446548	0.003576	19013, 13	100
GOTERM_	GO:00506	3	regulation	3	2.417344	0.003825	13617, 13	100
GOTERM_	GO:00081	9	metabolic	9	2.40886	0.003901	14387, 221	100
GOTERM_	GO:00510	3	positive re	3	2.367738	0.004288	13361, 11	100
GOTERM_	GO:00715	4	cellular res	4	2.341715	0.004553	14360, 11	100
GOTERM_	GO:00448	3	estrous cy	3	2.320979	0.004776	13983, 12	100
GOTERM_	GO:00071	9	cell adhesi	9	2.288819	0.005143	17136, 20	100
GOTERM_	GO:00603	3	face devel	3	2.276767	0.005287	26395, 10	100
GOTERM_	GO:00061	3	purine nuc	3	2.276767	0.005287	14450, 10	100
GOTERM_	GO:00091	3	nucleoside	3	2.276767	0.005287	22271, 10	100
GOTERM_	GO:00331	3	response t	3	2.276767	0.005287	20343, 12	100
GOTERM_	GO:00064	3	protein AD	3	2.276767	0.005287	21951, 74	100
GOTERM_	GO:00481	4	positive re	4	2.264451	0.005439	13649, 11	100
GOTERM_	GO:00060	4	glucose m	4	2.20988	0.006168	19013, 18	100
GOTERM_	GO:00359	3	cellular res	3	2.194997	0.006383	14254, 11	100
GOTERM_	GO:00355	8	intracellula	8	2.193	0.006412	12229, 13	100
GOTERM_	GO:00519	3	positive re	3	2.120786	0.007572	13649, 11	100
GOTERM_	GO:00331	4	positive re	4	2.108356	0.007792	17319, 18	100
GOTERM_	GO:00513	4	response t	4	2.092331	0.008085	26395, 221	100
GOTERM_	GO:00356	4	cellular res	4	2.092331	0.008085	13649, 141	100
GOTERM_	GO:00607	3	labyrinthin	3	2.052895	0.008853	15205, 11	100
GOTERM_	GO:00703	3	ERK1 and	3	2.052895	0.008853	26395, 26	100
GOTERM_	GO:00308	3	thyroid gla	3	2.052895	0.008853	26395, 10	100
GOTERM_	GO:20012	3	negative r	3	2.021012	0.009528	17395, 43	100
GOTERM_	GO:00486	4	positive re	4	2.015651	0.009646	13649, 15	100
GOTERM_	GO:00015	6	angiogene	6	1.994639	0.010124	16790, 14	100
GOTERM_	GO:00423	2	negative r	2	1.96176	0.01092	11539, 11	100
GOTERM_	GO:00464	2	dihydrofol	2	1.96176	0.01092	13361, 10	100
GOTERM_	GO:00702	2	negative r	2	1.96176	0.01092	11539, 11	100
GOTERM_	GO:00974	3	liver regen	3	1.960872	0.010943	13649, 221	100
GOTERM_	GO:00435	5	negative r	5	1.935388	0.011604	14360, 43	100
GOTERM_	GO:00425	3	positive re	3	1.932448	0.011683	433759, 1	100
GOTERM_	GO:00423	3	positive re	3	1.932448	0.011683	13649, 16	100
GOTERM_	GO:00551	10	oxidation-	10	1.928626	0.011786	19224, 221	100
GOTERM_	GO:00507	4	negative r	4	1.91693	0.012108	11539, 19	100
GOTERM_	GO:00316	3	lipopolysa	3	1.905022	0.012445	11651, 26	100
GOTERM_	GO:00335	3	response t	3	1.878531	0.013227	13983, 66	100
GOTERM_	GO:00713	3	cellular res	3	1.852915	0.014031	13649, 17	100
GOTERM_	GO:00702	2	protein po	2	1.786846	0.016336	21951, 11	100
GOTERM_	GO:00357	2	platelet-d	2	1.786846	0.016336	19246, 11	100
GOTERM_	GO:19038	2	positive re	2	1.786846	0.016336	26395, 13	100
GOTERM_	GO:00600	3	excitatory	3	1.780815	0.016565	11539, 11	100
GOTERM_	GO:00519	3	regulation	3	1.758216	0.01745	11540, 18	100
GOTERM_	GO:00086	3	carbohydr	3	1.758216	0.01745	246787, 11	100
GOTERM_	GO:00422	3	response t	3	1.736268	0.018354	13162, 15	100
GOTERM_	GO:00082	7	negative r	7	1.722492	0.018946	11539, 26	100
GOTERM_	GO:00059	3	glycogen r	3	1.714938	0.019278	14387, 11	100
GOTERM_	GO:00431	3	negative r	3	1.714938	0.019278	433759, 11	100

GOTERM_I	GO:00434	4	positive re	4	1.710717	0.019466	14254, 163	100
GOTERM_I	GO:00510	4	positive re	4	1.678382	0.020971	13983, 116	100
GOTERM_I	GO:00076	4	locomotor	4	1.667837	0.021486	13162, 143	100
GOTERM_I	GO:00902	2	positive re	2	1.663084	0.021723	12425, 124	100
GOTERM_I	GO:00336	2	regulation	2	1.663084	0.021723	13482, 114	100
GOTERM_I	GO:00701	2	response t	2	1.663084	0.021723	13649, 116	100
GOTERM_I	GO:00190	2	transforma	2	1.663084	0.021723	17395, 163	100
GOTERM_I	GO:00702	2	protein au	2	1.663084	0.021723	21951, 744	100
GOTERM_I	GO:00901	2	regulation	2	1.663084	0.021723	26395, 264	100
GOTERM_I	GO:00457	3	positive re	3	1.635194	0.023164	13649, 163	100
GOTERM_I	GO:00434	3	negative re	3	1.635194	0.023164	19246, 153	100
GOTERM_I	GO:00085	4	male gona	4	1.626773	0.023617	16337, 139	100
GOTERM_I	GO:00484	3	cell matura	3	1.598305	0.025217	12229, 152	100
GOTERM_I	GO:00434	3	pigmentat	3	1.598305	0.025217	13618, 221	100
GOTERM_I	GO:00071	3	epidermal	3	1.598305	0.025217	13649, 114	100
GOTERM_I	GO:00071	3	adenylate	3	1.598305	0.025217	11539, 147	100
GOTERM_I	GO:00350	3	somatic ste	3	1.580528	0.026271	15205, 109	100
GOTERM_I	GO:00485	3	spleen dev	3	1.580528	0.026271	11491, 113	100
GOTERM_I	GO:00059	5	carbohydr	5	1.574646	0.026629	14387, 141	100
GOTERM_I	GO:00069	4	chemotaxi	4	1.568302	0.027021	14254, 147	100
GOTERM_I	GO:00030	2	regulation	2	1.567349	0.02708	26395, 184	100
GOTERM_I	GO:00026	2	negative re	2	1.567349	0.02708	11539, 114	100
GOTERM_I	GO:00029	2	negative re	2	1.567349	0.02708	17319, 114	100
GOTERM_I	GO:00017	4	neuron mi	4	1.513246	0.030673	14360, 155	100
GOTERM_I	GO:00508	3	T cell rece	3	1.497589	0.031799	14360, 109	100
GOTERM_I	GO:00082	3	insulin rec	3	1.497589	0.031799	19246, 163	100
GOTERM_I	GO:00071	5	cell surface	5	1.490615	0.032314	14360, 168	100
GOTERM_I	GO:00509	2	regulation	2	1.489342	0.032408	13649, 133	100
GOTERM_I	GO:00033	2	brainstem	2	1.489342	0.032408	14450, 108	100
GOTERM_I	GO:00481	2	astrocyte a	2	1.489342	0.032408	13649, 115	100
GOTERM_I	GO:20003	2	positive re	2	1.489342	0.032408	433759, 17	100
GOTERM_I	GO:00019	2	intramemb	2	1.489342	0.032408	17390, 130	100
GOTERM_I	GO:00061	2	'de novo' I	2	1.489342	0.032408	14450, 108	100
GOTERM_I	GO:00082	5	cell prolife	5	1.484415	0.032778	26395, 136	100
GOTERM_I	GO:00315	3	actin cytos	3	1.48208	0.032955	19246, 124	100
GOTERM_I	GO:00485	4	rhythmic p	4	1.478276	0.033245	433759, 19	100
GOTERM_I	GO:00434	3	steroid ho	3	1.466898	0.034127	19013, 139	100
GOTERM_I	GO:00086	3	intrinsic ap	3	1.452031	0.035316	12044, 189	100
GOTERM_I	GO:00987	4	mitophagy	4	1.444593	0.035926	26395, 187	100
GOTERM_I	GO:00508	3	B cell rece	3	1.437466	0.03652	16818, 113	100
GOTERM_I	GO:00432	2	response t	2	1.423569	0.037708	11540, 120	100
GOTERM_I	GO:20006	2	regulation	2	1.423569	0.037708	26395, 264	100
GOTERM_I	GO:00600	2	Sertoli cell	2	1.423569	0.037708	13983, 139	100
GOTERM_I	GO:00459	2	negative re	2	1.423569	0.037708	11539, 153	100
GOTERM_I	GO:00604	2	trachea fo	2	1.423569	0.037708	26395, 264	100
GOTERM_I	GO:00311	3	organ rege	3	1.423194	0.03774	108147, 11	100
GOTERM_I	GO:00458	8	positive re	8	1.411761	0.038747	26395, 433	100
GOTERM_I	GO:00015	3	ovarian fol	3	1.409202	0.038976	17390, 139	100
GOTERM_I	GO:00425	3	response t	3	1.395483	0.040227	16818, 173	100
GOTERM_I	GO:00066	7	lipid metal	7	1.394777	0.040292	19224, 190	100
GOTERM_I	GO:00072	3	phospholip	3	1.368821	0.042774	13618, 139	100
GOTERM_I	GO:00158	2	monoamir	2	1.36675	0.042978	13162, 205	100
GOTERM_I	GO:00328	2	regulation	2	1.36675	0.042978	26395, 264	100
GOTERM_I	GO:00108	2	positive re	2	1.36675	0.042978	14254, 139	100
GOTERM_I	GO:00600	2	relaxation	2	1.36675	0.042978	11539, 115	100
GOTERM_I	GO:20010	2	regulation	2	1.36675	0.042978	11350, 172	100
GOTERM_I	GO:00023	2	myeloid pr	2	1.36675	0.042978	109880, 16	100
GOTERM_I	GO:00026	2	positive re	2	1.36675	0.042978	17319, 309	100

GOTERM_I	GO:004868	2	positive re	2	1.36675	0.042978	17319, 109		100
GOTERM_I	GO:009028	2	negative re	2	1.36675	0.042978	15205, 114		100
GOTERM_I	GO:003581	2	negative re	2	1.36675	0.042978	11539, 167		100
GOTERM_I	GO:000169	2	gastric acid	2	1.36675	0.042978	12425, 124		100
GOTERM_I	GO:003209	3	negative re	3	1.355861	0.04407	19013, 114		100
GOTERM_I	GO:003292	3	circadian r	3	1.355861	0.04407	433759, 19		100
GOTERM_I	GO:000854	3	epidermis	3	1.343138	0.04538	13649, 190		100
GOTERM_I	GO:004594	11	positive re	11	1.340457	0.045661	21951, 136		100
GOTERM_I	GO:004881	3	neuron pro	3	1.330645	0.046704	26395, 136		100
GOTERM_I	GO:005072	3	positive re	3	1.330645	0.046704	13617, 136		100
GOTERM_I	GO:000854	3	visual learr	3	1.330645	0.046704	109880, 15		100
GOTERM_I	GO:007136	3	cellular res	3	1.318373	0.048043	13649, 163		100
GOTERM_I	GO:001097	3	negative re	3	1.318373	0.048043	17136, 114		100
GOTERM_I	GO:001406	3	positive re	3	1.318373	0.048043	14360, 203		100
GOTERM_I	GO:005079	2	activated T	2	1.316768	0.04822	14360, 113		100
GOTERM_I	GO:200027	2	negative re	2	1.316768	0.04822	30955, 187		100
GOTERM_I	GO:004574	2	positive re	2	1.316768	0.04822	11539, 114		100
GOTERM_I	GO:002198	2	adenohypo	2	1.316768	0.04822	13162, 152		100
GOTERM_I	GO:007019	2	protein loc	2	1.316768	0.04822	21951, 744		100
GOTERM_I	GO:003243	3	positive re	3	1.306315	0.049395	14466, 116		100

Pop Hits	Pop Total	Fold Enrich	Bonferroni	Benjamini	FDR
339	18082	10.13445	4.82E-10	4.82E-10	4.59E-10
65	18082	27.81846	1.10E-07	5.51E-08	5.24E-08
576	18082	6.278472	3.94E-07	1.31E-07	1.25E-07
183	18082	11.85705	7.15E-06	1.51E-06	1.43E-06
612	18082	5.613693	7.54E-06	1.51E-06	1.43E-06
77	18082	21.13481	1.36E-05	2.27E-06	2.15E-06
566	18082	5.430989	1.02E-04	1.46E-05	1.39E-05
148	18082	12.21757	1.74E-04	2.04E-05	1.94E-05
197	18082	10.09655	1.83E-04	2.04E-05	1.94E-05
188	18082	9.618085	0.001319	1.32E-04	1.26E-04
100	18082	14.4656	0.001827	1.66E-04	1.58E-04
542	18082	5.004244	0.00213	1.78E-04	1.69E-04
41	18082	26.46146	0.004135	3.19E-04	3.03E-04
227	18082	7.965639	0.006231	4.46E-04	4.25E-04
173	18082	9.406821	0.00707	4.73E-04	4.50E-04
184	18082	8.844457	0.011108	6.98E-04	6.64E-04
192	18082	8.475938	0.015131	8.97E-04	8.53E-04
55	18082	19.72582	0.017942	9.93E-04	9.45E-04
27	18082	33.48519	0.019406	9.93E-04	9.45E-04
570	18082	4.441193	0.01972	9.93E-04	9.45E-04
265	18082	6.823396	0.021488	9.93E-04	9.45E-04
335	18082	5.937373	0.021701	9.93E-04	9.45E-04
203	18082	8.01665	0.022591	9.93E-04	9.45E-04
582	18082	4.349622	0.024508	0.001034	9.83E-04
344	18082	5.782035	0.027188	0.001103	0.001049
62	18082	17.49871	0.032147	0.001257	0.001195
117	18082	10.81829	0.062416	0.002387	0.00227
38	18082	23.79211	0.075689	0.002811	0.002674
16	18082	45.205	0.115894	0.004116	0.003915
43	18082	21.02558	0.120903	0.004116	0.003915
3	18082	180.82	0.121091	0.004116	0.003915
133	18082	9.516842	0.123399	0.004116	0.003915
45	18082	20.09111	0.142966	0.004675	0.004447
19	18082	38.06737	0.189919	0.006194	0.005892
4	18082	135.615	0.226815	0.007349	0.00699
54	18082	16.74259	0.270482	0.008759	0.008332
22	18082	32.87636	0.281636	0.008939	0.008503
23	18082	31.44696	0.315381	0.00997	0.009483
24	18082	30.13667	0.350344	0.011058	0.010518
399	18082	4.53183	0.391024	0.012397	0.011792
1255	18082	2.593434	0.469872	0.014653	0.013938
6	18082	90.41	0.471963	0.014653	0.013938
6	18082	90.41	0.471963	0.014653	0.013938
65	18082	13.90923	0.475274	0.014653	0.013938
7	18082	77.49429	0.589717	0.019792	0.018826
31	18082	23.33161	0.606153	0.02025	0.019262
75	18082	12.05467	0.670622	0.023369	0.022229
33	18082	21.91758	0.67442	0.023369	0.022229
199	18082	6.360503	0.683409	0.023434	0.02229
8	18082	67.8075	0.693874	0.023434	0.02229
133	18082	8.157293	0.697478	0.023434	0.02229
78	18082	11.59103	0.723953	0.024743	0.023535
38	18082	19.03368	0.817564	0.032082	0.030517
86	18082	10.51279	0.843357	0.033717	0.032072
10	18082	54.246	0.848834	0.033717	0.032072
10	18082	54.246	0.848834	0.033717	0.032072
41	18082	17.64098	0.880546	0.03725	0.035433

90	18082	10.04556	0.88853	0.037799	0.035955
45	18082	16.07289	0.938252	0.047153	0.044852
14	18082	38.74714	0.976925	0.061763	0.058749
50	18082	14.4656	0.977004	0.061763	0.058749
51	18082	14.18196	0.98155	0.06431	0.061172
108	18082	8.371296	0.986058	0.067723	0.064418
261	18082	4.849579	0.989471	0.07104	0.067573
16	18082	33.90375	0.992822	0.073559	0.069969
16	18082	33.90375	0.992822	0.073559	0.069969
16	18082	33.90375	0.992822	0.073559	0.069969
56	18082	12.91571	0.994555	0.076525	0.072791
17	18082	31.90941	0.996214	0.080662	0.076726
463	18082	3.51486	0.996609	0.081078	0.077122
18	18082	30.13667	0.998074	0.087875	0.083587
61	18082	11.85705	0.998692	0.092006	0.087516
19	18082	28.55053	0.999056	0.095183	0.090539
485	18082	3.355423	0.999448	0.09738	0.092628
20	18082	27.123	0.999553	0.09738	0.092628
20	18082	27.123	0.999553	0.09738	0.092628
20	18082	27.123	0.999553	0.09738	0.092628
20	18082	27.123	0.999553	0.09738	0.092628
20	18082	27.123	0.999553	0.09738	0.092628
65	18082	11.12738	0.999642	0.098929	0.094101
68	18082	10.63647	0.999877	0.110789	0.105383
22	18082	24.65727	0.99991	0.112405	0.10692
400	18082	3.6164	0.999914	0.112405	0.10692
24	18082	22.6025	0.999984	0.131159	0.124759
74	18082	9.774054	0.999989	0.133379	0.126871
75	18082	9.643733	0.999993	0.135211	0.128613
75	18082	9.643733	0.999993	0.135211	0.128613
26	18082	20.86385	0.999998	0.143128	0.136144
26	18082	20.86385	0.999998	0.143128	0.136144
26	18082	20.86385	0.999998	0.143128	0.136144
27	18082	20.09111	0.999999	0.152339	0.144905
80	18082	9.041	0.999999	0.152554	0.14511
239	18082	4.539414	1	0.158395	0.150666
2	18082	180.82	1	0.164142	0.156132
2	18082	180.82	1	0.164142	0.156132
2	18082	180.82	1	0.164142	0.156132
29	18082	18.70552	1	0.164142	0.156132
160	18082	5.650625	1	0.169792	0.161506
30	18082	18.082	1	0.169792	0.161506
30	18082	18.082	1	0.169792	0.161506
676	18082	2.674852	1	0.169792	0.161506
87	18082	8.313563	1	0.172716	0.164288
31	18082	17.49871	1	0.175794	0.167216
32	18082	16.95188	1	0.185054	0.176024
33	18082	16.43818	1	0.194428	0.18494
3	18082	120.5467	1	0.220086	0.209347
3	18082	120.5467	1	0.220086	0.209347
3	18082	120.5467	1	0.220086	0.209347
36	18082	15.06833	1	0.221116	0.210327
37	18082	14.66108	1	0.228731	0.217569
37	18082	14.66108	1	0.228731	0.217569
38	18082	14.27526	1	0.238439	0.226804
384	18082	3.296198	1	0.243908	0.232006
39	18082	13.90923	1	0.243908	0.232006
39	18082	13.90923	1	0.243908	0.232006

104	18082	6.954615	1	0.244168	0.232253
107	18082	6.759626	1	0.254893	0.242455
108	18082	6.697037	1	0.254893	0.242455
4	18082	90.41	1	0.254893	0.242455
4	18082	90.41	1	0.254893	0.242455
4	18082	90.41	1	0.254893	0.242455
4	18082	90.41	1	0.254893	0.242455
4	18082	90.41	1	0.254893	0.242455
4	18082	90.41	1	0.254893	0.242455
43	18082	12.61535	1	0.267485	0.254432
43	18082	12.61535	1	0.267485	0.254432
112	18082	6.457857	1	0.270574	0.257371
45	18082	12.05467	1	0.280083	0.266416
45	18082	12.05467	1	0.280083	0.266416
45	18082	12.05467	1	0.280083	0.266416
45	18082	12.05467	1	0.280083	0.266416
46	18082	11.79261	1	0.285519	0.271586
46	18082	11.79261	1	0.285519	0.271586
206	18082	4.388835	1	0.285519	0.271586
118	18082	6.129492	1	0.285519	0.271586
5	18082	72.328	1	0.285519	0.271586
5	18082	72.328	1	0.285519	0.271586
5	18082	72.328	1	0.285519	0.271586
124	18082	5.832903	1	0.31861	0.303062
51	18082	10.63647	1	0.31861	0.303062
51	18082	10.63647	1	0.31861	0.303062
219	18082	4.128311	1	0.31861	0.303062
6	18082	60.27333	1	0.31861	0.303062
6	18082	60.27333	1	0.31861	0.303062
6	18082	60.27333	1	0.31861	0.303062
6	18082	60.27333	1	0.31861	0.303062
6	18082	60.27333	1	0.31861	0.303062
6	18082	60.27333	1	0.31861	0.303062
220	18082	4.109545	1	0.319662	0.304064
52	18082	10.43192	1	0.319662	0.304064
128	18082	5.650625	1	0.320339	0.304707
53	18082	10.23509	1	0.326679	0.310738
54	18082	10.04556	1	0.335847	0.319458
132	18082	5.479394	1	0.33943	0.322866
55	18082	9.862909	1	0.34107	0.324427
7	18082	51.66286	1	0.34107	0.324427
7	18082	51.66286	1	0.34107	0.324427
7	18082	51.66286	1	0.34107	0.324427
7	18082	51.66286	1	0.34107	0.324427
7	18082	51.66286	1	0.34107	0.324427
56	18082	9.686786	1	0.34107	0.324427
576	18082	2.511389	1	0.347915	0.330938
57	18082	9.516842	1	0.347915	0.330938
58	18082	9.352759	1	0.353297	0.336057
459	18082	2.757603	1	0.353297	0.336057
60	18082	9.041	1	0.353297	0.336057
8	18082	45.205	1	0.353297	0.336057
8	18082	45.205	1	0.353297	0.336057
8	18082	45.205	1	0.353297	0.336057
8	18082	45.205	1	0.353297	0.336057
8	18082	45.205	1	0.353297	0.336057
8	18082	45.205	1	0.353297	0.336057
8	18082	45.205	1	0.353297	0.336057

8	18082	45.205	1	0.353297	0.336057
8	18082	45.205	1	0.353297	0.336057
8	18082	45.205	1	0.353297	0.336057
8	18082	45.205	1	0.353297	0.336057
61	18082	8.892787	1	0.358219	0.340739
61	18082	8.892787	1	0.358219	0.340739
62	18082	8.749355	1	0.365421	0.347589
995	18082	1.999015	1	0.365421	0.347589
63	18082	8.610476	1	0.365421	0.347589
63	18082	8.610476	1	0.365421	0.347589
63	18082	8.610476	1	0.365421	0.347589
64	18082	8.475938	1	0.365421	0.347589
64	18082	8.475938	1	0.365421	0.347589
64	18082	8.475938	1	0.365421	0.347589
9	18082	40.18222	1	0.365421	0.347589
9	18082	40.18222	1	0.365421	0.347589
9	18082	40.18222	1	0.365421	0.347589
9	18082	40.18222	1	0.365421	0.347589
9	18082	40.18222	1	0.365421	0.347589
65	18082	8.345538	1	0.372383	0.354212
